# Machine Learning-Based Analysis of MR Multiparametric Radiomics for the Subtype Classification of Breast Cancer

**DOI:** 10.3389/fonc.2019.00505

**Published:** 2019-06-14

**Authors:** Tianwen Xie, Zhe Wang, Qiufeng Zhao, Qianming Bai, Xiaoyan Zhou, Yajia Gu, Weijun Peng, He Wang

**Affiliations:** ^1^Department of Radiology, Fudan University Shanghai Cancer Center, Shanghai, China; ^2^Department of Oncology, Fudan University Shanghai Cancer Center, Shanghai, China; ^3^Human Phenome Institute, Fudan University, Shanghai, China; ^4^Shanghai Center for Mathematical Sciences, Fudan University, Shanghai, China; ^5^Department of Radiology, Longhua Hospital, Shanghai University of Traditional Chinese Medicine, Shanghai, China; ^6^Department of Pathology, Fudan University Shanghai Cancer Center, Shanghai, China; ^7^Institute of Science and Technology for Brain-Inspired Intelligence, Fudan University, Shanghai, China

**Keywords:** radiomics, breast cancer, immunohistochemical subtypes, dynamic contrast-enhanced imaging, diffusion-weighted imaging, machine learning

## Abstract

**Objective:** To investigate whether machine learning analysis of multiparametric MR radiomics can help classify immunohistochemical (IHC) subtypes of breast cancer.

**Study design:** One hundred and thirty-four consecutive patients with pathologically-proven invasive ductal carcinoma were retrospectively analyzed. A total of 2,498 features were extracted from the DCE and DWI images, together with the new calculated images, including DCE images changing over six time points (DCE_sequential_) and DWI images changing over three *b*-values (DWI_sequential_). We proposed a novel two-stage feature selection method combining traditional statistics and machine learning-based methods. The accuracies of the 4-IHC classification and triple negative (TN) vs. non-TN cancers were assessed.

**Results:** For the 4-IHC classification task, the best accuracy of 72.4% was achieved based on linear discriminant analysis (LDA) or subspace discrimination of assembled learning in conjunction with 20 selected features, and only small dependent emphasis of Kendall-tau-b for sequential features, based on the DWI_sequential_ with the LDA model, yielding an accuracy of 53.7%. The linear support vector machine (SVM) and medium k-nearest neighbor using eight features yielded the highest accuracy of 91.0% for comparing TN to non-TN cancers, and the maximum variance for DWI_sequential_ alone, together with a linear SVM model, achieved an accuracy of 83.6%.

**Conclusions:** Whole-tumor radiomics on MR multiparametric images, DCE images changing over time points, and DWI images changing over different *b*-values provide a non-invasive analytical approach for breast cancer subtype classification and TN cancer identification.

## Introduction

Breast cancer is a heterogeneous disease with diverse clinical manifestations, treatment responses, and survival outcomes (1). The immunohistochemical (IHC) subtypes based on the expression of the estrogen and progesterone receptor, the detection of overexpression of the human epidermal growth factor receptor 2 (HER2) oncogene, and Ki-67 labeling index, are routinely used to identify tumor subtypes with different clinical outcomes and responses to therapy, including Luminal A cancer, Luminal B cancer, HER2-positive cancer, and triple negative (TN) cancer (2). HER2-positive breast cancers are more likely to have a pathologic complete response (pCR) to neoadjuvant chemotherapy, whereas reduced pCR rates are demonstrated in Luminal-type breast cancers (2, 3). Patients with TN breast cancer have a relatively high death rate due to the aggressive features of this subtype and lack of effective targeted therapy (4). Therefore, it is important to identify the subtypes to select the appropriate therapy and predict the therapeutic response (5).

Multiparametric MR imaging, including dynamic contrast-enhanced (DCE) imaging and diffusion-weighted imaging (DWI), has excellent sensitivity and good specificity for breast cancer diagnosis and plays an important role in the preoperative staging and subsequent choice of appropriate therapy. If DCE imaging and DWI can be used to differentiate breast cancer subtypes, this would provide a complementary method for constructing the IHC profile and therefore assist in treatment planning. Several authors have described the breast cancer subtypes with the BI-RADS lexicon on MRI (6–8), but inter- and intra-observer variability continues to exist among radiologists (9, 10).

Beyond visual interpretation by radiologists, there is more quantitative diagnostic information about the tumor hidden in thousands of acquired images. Radiomics (referring to computational algorithms) used extracted imaging texture features to serve as noninvasive biomarkers that could predict breast cancer subtypes (10). Multiple studies have used the texture analysis of DCE images for the subtype classification of breast cancer or the identification of TN cancer (11, 12). Grimm et al. demonstrated a correlation between Luminal subtypes of breast cancer and texture features on DCE images (13). Agner et al. used the morphologic and texture features extracted from the whole tumor on early postcontrast images in conjunction with an SVM classifier to identify TN cancer (14). Several studies have proposed the histogram or textural features extracted from DWI for subtype differentiation (15, 16). However, no study has attempted to investigate the texture analysis based radiomics of combining DCE imaging and DWI in the subtype classification of breast cancer.

The purpose of this present study was to evaluate the performance of the MR multiparametric radiomics model to differentiate among Luminal A cancer, Luminal B cancer, HER2-positive cancer, and TN breast cancer using DCE imaging and DWI. Furthermore, we investigated whether the radiomic model could identify the subtype of the worst clinical outcome (TN breast cancer) from other subtypes.

## Materials and Methods

### Study Population

Between February 2016 and May 2017, 190 consecutive patients with core needle biopsy-proven invasive ductal carcinoma (IDC) were enrolled in the study. All the patients underwent preoperative MRI examinations with DCE and DWI sequences. Patients with a prior history of malignancy (*N* = 5), those treated with neoadjuvant chemotherapy before MR examination (*N* = 31), and those with lesions smaller than 1 cm (*N* = 16) were excluded. Our database also excluded cases that had poor fat saturation on DWI (*N* = 3) or discordance in the number of slices among the postcontrast sequences (*N* = 1). Two radiologists with 2- and 8-years' experience in breast MR imaging, respectively, who were blinded to the pathologic results but were aware of the IDC diagnosis, reviewed the MR images. For 22 patients with multicentric or multifocal tumors, the largest tumor was selected for analysis based on the first post-contrast DCE images. A total of 134 tumors from 134 women (mean age, 51.2 years; age range, 24–84 years) were ultimately evaluated.

The immunohistochemical subtype of breast cancer was classified as Luminal A (ER- and/or PR-positive, HER2-negative, and Ki-67 < 14%), Luminal B (ER- and/or PR-positive, HER2-negative, and Ki-67 ≥ 14%, or ER- and/or PR-positive, HER2-positive, irrespective of Ki-67 expression), HER2-positive (ER- and PR-negative, HER2-positive), and TN (ER-negative, PR-negative, and HER2-negative) (5).

This retrospective study was approved by our institutional review board, which waived informed consent.

### MR Imaging

All the MR scans were performed using a 3.0T MAGNETOM Skyra system (Siemens Healthcare, Erlangen, Germany) with a 16-channel phased-array breast coil, with patients in the prone position. The breast MRI examinations included a transverse fat-suppressed T2-weighted (TR/TE, 3,570/69 ms) sequence and a transverse T1-weighted (TR/TE, 5.4/2.4 ms) sequence.

Before contrast injection, DWI was performed in the transverse plane covering both breasts at the position of the tumor using a single-shot echo-planar imaging sequence with the following parameters: TR/TE, 3,000/54 ms; flip angle, 90°; field of view, 340 × 150 – 280 mm^2^; matrix, 220 × 220; slice thickness, 6 mm; 3 *b*-values, 50, 400, and 800 s/mm^2^, with the number of averages 3, 4, and 5, respectively; rate 3 GRAPPA acceleration. The total acquisition time was 2:09 min.

DCE-MRI was performed using a 3D T1-weighted fat-suppressed, fast spoiled gradient-echo sequence (TR/TE 4.5/1.6 ms; flip angle, 10°; bandwidth, 380 Hz/Pixel) with one pre-contrast and five consecutive post-contrast dynamic series after a bolus injection of 0.1 mmol/L of gadopentetate dimeglumine (Magnevist; Bayer Schering Pharma, Berlin, Germany) per kilogram of body weight, injected at a rate of 1.5 mL/s. Image acquisition in the transverse plane lasted for 60 s per volumetric acquisition with slice thickness and was 1.5 mm with no gap and a matrix of 384 × 384.

### Multiparametric MR Radiomics

**Figure 1:**
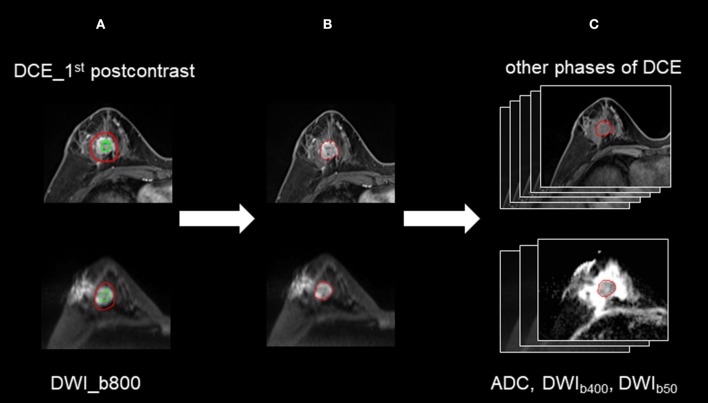
The process for 3D segmentation. The segmentation process comprised three steps: **(A)** The foreground and background seed points were manually drawn inside and outside the tumor on the DWI images with a *b*-value of 800 s/mm^2^ (DWI_b800_) and the first postcontrast of the DCE images, respectively. **(B)** The whole-tumor segmentations on DWI_b800_ and the first postcontrast of the DCE images were executed with a semiautomatic algorithm. **(C)** For DWI, 3D segmented contouring that was created on DWI_b800_ was propagated to the DWI images with other *b*-values. For DCE, 3D segmented contouring that was created on the first postcontrast images was propagated to the precontrast and four postcontrast phases of the DCE images.

The semi-automated segmentation of whole tumor on the DWI and DCE images was conducted by the radiologist using the prototype MR Multiparameter Analysis software (Siemens Healthcare, Erlangen, Germany). The segmentation process was composed of three steps: (a) The seed points were manually drawn inside the tumor (including the necrotic regions) and outside the tumor on DWI images with a *b*-value of 800 s/mm^2^ (DWI_b800_) and the first postcontrast of DCE images, respectively; (b) The 3D segmentation of the whole tumor was executed (based on these seed points) with a random-walker algorithm (17). We performed manual adjustments in 9 of 134 women for DWI_b800_ and in 46 of 134 women for the first postcontrast of DCE images; and (c) For DWI, 3D segmented contouring created on DWI_b800_ was propagated to the DWI images with other *b*-values. For DCE, 3D segmented contouring created on the first postcontrast images were propagated to pre-contrast and the other four post-contrast phases of DCE images. Finally, 3D segmentation of the whole tumor from nine different imaging series (DWI MR images with three different *b*-values, and six DCE images) were saved. The progress for 3D segmentation is described in Figure 1.

The 3D segmentation from the nine-image series were loaded onto a personal laptop for further texture analysis. The radiomics features were comprised of five parts: (a) Shape features on DCE (DCE_shape_): The fourteen shape-based features were calculated using the first postcontrast DCE images; (b) Texture features based on DCE images (DCE_texture_): The 92 texture features (18) features, were calculated on six time series individually, yielding 552 features; (c) Sequential features based on DCE time series (DCE_sequential_): To characterize the textural changes on DCE images over time serials, we measured ten new sequential features for each texture feature described in group b (Supplemental Material 1). The first six features, including mean, variance, kurtosis, skewness, energy, and entropy, were extracted from each individual subject. The other four features, including Kendall-tau-b, conservation, stability, and dispersion, were calculated from the interactive information between the current subject and the remainder of the subjects. Therefore, a total of 920 DCE_sequential_ features were extracted from 92 texture features; (d) Texture features based on DWI images (DWI_texture_): The same 92 texture features with group b were calculated on DWI images with three *b*-values set to yield 276 features; and (e) Sequential features based on DWI images (DWI_sequential_): Like group c, to characterize the textural changes on DWI images over different *b*-values, we measured eight sequential features for each texture feature. Kurtosis and entropy were not measured because of the limited number of *b*-values. Therefore, 736 DWI_sequential_ features were extracted. A total of 2,498 features were extracted to obtain a classification model. Texture extraction was applied to the multiparametric images using PyRadiomics package (18) in the Python software (v. 3.6, Python Software Foundation, https://www.python.org/).

### Feature Selection

To reduce the dimensionality of the feature space, we proposed a two-staged method combining traditional statistics and machine learning-based feature selection.

**Figure 2:**
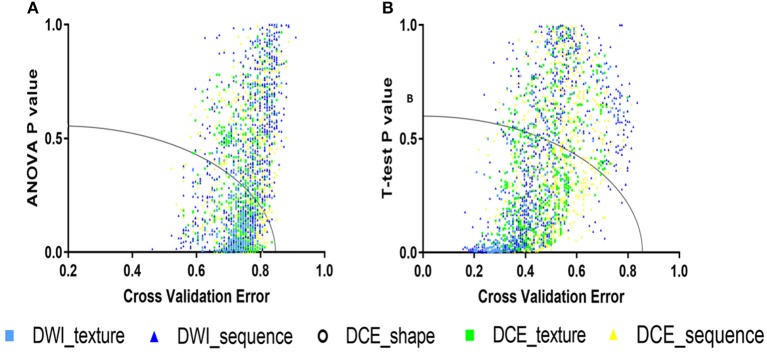
The accepted domains for coarse feature selection. **(A)** For the 4-IHC classification, the *p*-value for the analysis of variance (ANOVA) and cross-validation error were set to 0.6 and 0.85, respectively. **(B)** For the TN vs. non-TN cancers, the *t*-test *p*-value and cross-validation error were set to 0.54 and 0.76, respectively.

For coarse feature selection, the features that were considered to be noisy were excluded. The purpose of the coarse feature selection was to prevent the resulting model from becoming a linear combination of noises. Regarding the 4-IHC classification tasks of breast cancer, the one-way analysis of variance (ANOVA) and cross-validation (CV) were performed for every radiomics feature. The *t*-test and CV for each radiomics feature were performed for TN vs. non-TN cancers. The feature that had both a small *p*-value and a small cross-validation error rate was informative. The feasible domain for excluding noisy features was a strong convex set, which reduced a high dimensionality of complex data sets to a two-dimensional space (19). In addition, the ellipse was chosen as the acceptance domain. Based on this idea, the accepted domain equation was set as follows:

(1) $$\begin{array}{r} \frac{{\left(p\text{\_}value\right)}^{2}}{a^{2}}+\frac{{\left(CVerrorrate\right)}^{2}}{b^{2}}<1 \end{array}$$

where a and b are acceptance factors. In order to reduce approximately half of the features, we set a and b to 0.6 and 0.85, respectively, for the 4-IHC classification after an ergodic process by an interval of 0.01. For TN vs. non-TN cancers, a and b were set to 0.54 and 0.76, respectively (Figure 2).

For fine feature selection, we proposed a novel model by combing the lasso regression, ridge regression, and elastic net to select the features. For the 4-IHC classification, lasso regression, ridge regression, and elastic net were utilized separately to rank the selected features from the coarse feature selection. The ranks from these three methods were summed up as a score for each feature. The top 40 features with the lowest scores were selected, and 20 features that were correlated with other features (the absolute value of the Pearson correlation index > 0.4) were subsequently removed manually to reduce the collinearity of feature combinations. Using the same selection method as the 4-IHC classification, a total of eight features were selected for TN vs. non-TN.

### Machine Learning-Based Classification

The best models for the 4-IHC classification and TN vs. non-TN cancers were selected from three decision tree classifiers, two discriminant analysis classifiers, six SVM classifiers, four k-nearest neighbor (KNN) classifiers, and five ensemble learning classifiers (20–24). The three decision tree classifiers were fine tree, medium tree, and coarse tree. The two discriminant analysis classifiers were LDA and quadratic discriminant analysis (QDA). The six SVM classifiers included the linear, quadratic, cubic, fine gaussian, medium gaussian, and coarse gaussian. The four KNN classifiers were fine KNN, medium KNN, coarse KNN, and weighted KNN. The five ensemble learning classifiers included subspace discriminant, subspace KNN, the AdaBoost algorithm with decision tree, the bootstrap-aggregated (bagged) tree algorithm with decision tree, and the RUSBoost algorithm with decision tree.

**Figure 3:**
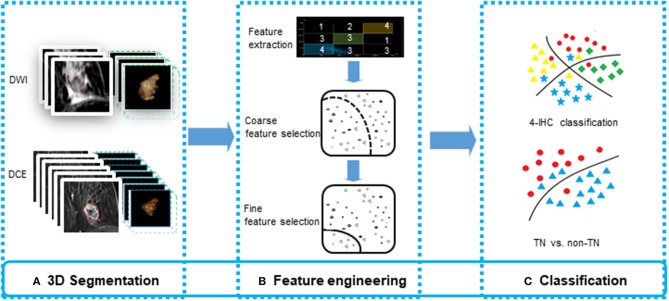
The flowchart for feature engineering of multiparametric MR radiomics. **(A)** The whole-tumor segmentations from a total of 10 sequence images were executed. **(B)** A total of 2,498 features were extracted, and a two-stage feature selection method was subsequently performed. **(C)** Machine learning-based classifiers were used for the 4-IHC classification and TN vs. non-TN cancers.

All the machine learning models were conducted using the 5-fold cross validation, whereby 20% of the data were used to test the model created by the other 80% of the data. The procedure was repeated for ten rounds to average the estimates of performance. The accuracy for two classification tasks was assessed for the models. To prove the superiority of our fine feature selection method, the classification accuracy combining lasso regression, ridge regression, and elastic net, and the classification accuracy using any single one of these three methods, were also assessed. The feature selection and machine learning-based classification was achieved using the Statistics and Machine Learning Toolbox in MatLab (v. R2018a; MathWorks, Natick, MA). The flowchart for multiparametric MR radiomics is described in Figure 3.

## Results

### Clinical Data

The patient demographic and cancer characteristics are shown in Table 1. Of the 134 cancers, the numbers of Luminal A, Luminal B, HER2-positive, and TN cancers were 26, 68, 18, and 22, respectively.

**Table 1:** Clinicopathologic characteristics of patients.

**Parameter**	**Luminal A**	**Luminal B**	**HER2-positive**	**TN**
Age (y)^*^	26	68	18	22
Menopausal status				
Peri- or postmenopausal	11	29	8	14
Premenopausal	15	38	10	8
Unknown	0	1	0	0
Tumor size (mm^3^)^*^	18 (11–47)	25 (10–80)	21 (10–43)	24 (13–39)
Axial lymph nodes				
Negative	21	30	7	13
Positive	5	38	11	9

*HER2, human epidermal growth factor receptor 2; TN, triple negative*.

* *Data for continuous variables are means ± standard deviation, with medians in parentheses*.

### Performance of the Machine Learning-Based Classification

**Figure 4:**
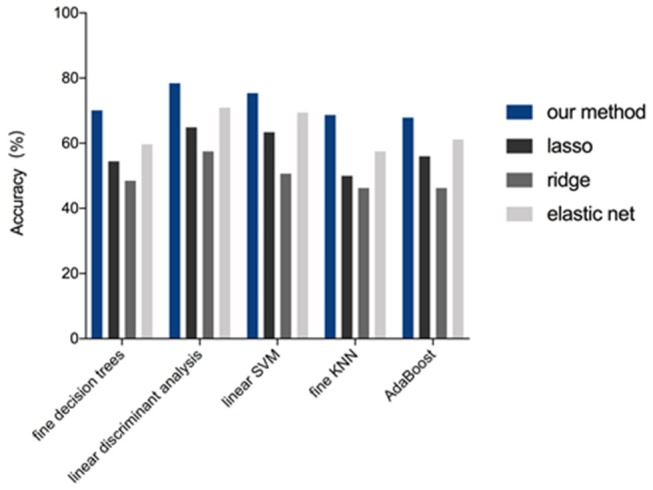
Performance of feature selection methods for the 4-IHCclassification. The accuracy for the four methods (our method, lasso, ridge, and elastic net) was compared while using five representative machine-learning models (fine decision trees, linear discriminant analysis, linear SVM, fine KNN, and AdaBoost algorithm with decision tree of ensemble learning).

**Figure 5:**
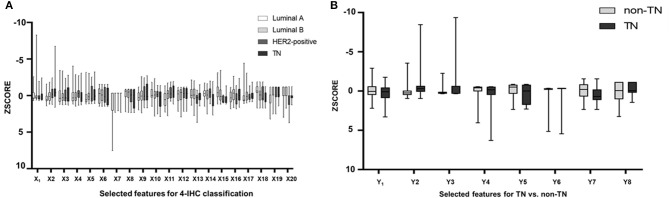
Box plots showed z-score value distribution of every selected feature for all patients in the 4-IHC classification **(A)** and TN vs. non-TN **(B)**.

**Figure 6:**
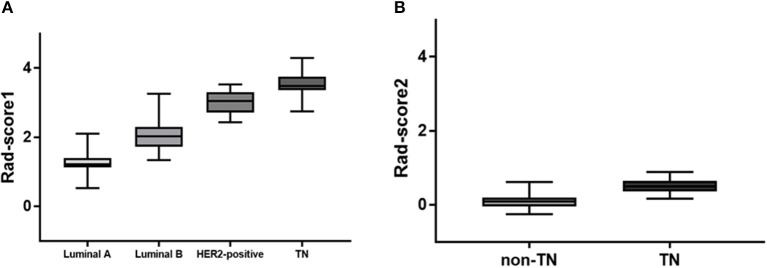
Rad-score box plots. **(A)** Rad-score box plot for 4-IHC classification (*p* = 4.2355e-46). **(B)** Rad-score box plot for TN vs. non-TN (*p* = 3.9100e-17).

Of 2,498 features, a total of 1,292 for the 4-IHC classification and 1,555 for the TN vs. non-TN cancers were chosen after coarse feature selection. Fine feature selection was conducted to reduce the feature sets to 20 features for the 4-IHC classification and eight for the TN vs. non-TN cancers (Supplementary Materials 2, 3). Z-score value distribution of every selected feature for all the patients in two classification tasks is displayed in Figure 4. As shown in Figure 5, our fine feature selection combining the lasso regression, ridge regression, and elastic net had higher accuracy compared with the use of any single one of these three methods. The Rad-score formulas for the two classification tasks were calculated via a linear combination of selected features that were weighted by the respective coefficients (Supplementary Materials 4, 5, and Figure 6).

The results for model performance of the two classification tasks are listed in Table 2. For the 4-IHC classification task, the accuracy of the dataset for different classifiers ranged from 50.7 to 72.4%. The best models with the highest accuracy were the LDA and subspace discriminant of ensemble learning. Notably, only a small dependence emphasis of Kendall-tau-b feature for DWI_sequential_ with the LDA model yielded the highest accuracy of 53.7%.

**Table 2:** Model performance for the test datasets of two classification tasks.

	**Classifiers**	**Accuracy (4-IHC classification)**	**Accuracy (TN vs. non-TN)**
Decision tree	Fine tree	69.4%	87.3%
	Medium tree	69.4%	87.3%
	Coarse tree	64.9%	88.8%
Discriminant analysis	LDA	72.4%	90.3%
	QDA	70.9%	90.3%
SVM	Linear	67.9%	91.0%
	Quadratic	69.4%	89.6%
	Cubic	59.7%	72.4%
	Fine gaussian	68.7%	87.3%
	Median gaussian	68.7%	89.6%
	Coarse gaussian	70.1%	88.1%
KNN	Fine	69.4%	87.3%
	Medium	64.9%	91.0%
	Coarse	50.7%	83.6%
	Weighted	70.1%	88.1%
Ensemble learning	Subspace discriminant	72.4%	90.3%
	Subspace KNN	69.4%	87.3%
	AdaBoost tree	67.2%	83.6%
	Bagged tree	70.1%	88.1%
	RUSBoost tree	58.2%	85.8%

*LDA, linear discriminant analysis; QDA, quadratic discriminant analysis; SVM, support vector machines; KNN, k-nearest neighbor; Bagged, bootstrap-aggregated; IHC, Immunohistochemical; TN, triple negative*.

When comparing the TN to the non-TN cancers, the accuracy of the test dataset ranged from 72.4 to 91.0%. The linear SVM and medium KNN each yielded the highest accuracy of 91.0%. With only the maximum of variance feature for DWI_sequential_ with the linear SVM model, we obtained an accuracy of 83.6%.

## Discussion

We investigated whether different breast cancer subtypes could be differentiated on multiparametric MR radiomics. Texture features extracted from DCE- and DWI-related original images, particularly those sequential features changing over time points or several *b*-values, could quantify the heterogenous differences between the IHC subtypes. Although this is early work, multiparametric MR radiomics has the potential to provide a non-invasive approach and further insight into tumor imaging phenotypes. Moreover, the methodology may assist in the differentiation of details of subtypes that are imperceptible to the human eye and is also free of inter- or intra-observer variability (9).

Multiparametric imaging assessing tumor heterogeneity has recently been extended to help identify the subtype of breast cancer (25–28). The combined MR texture features could make the process of subtype classification quantitative and yield increased accuracy. Waugh et al. extracted 220 GLCM features in 72 IDC patients and found that the accuracy for subtype classification was 57.2% using all features and 43.6% using only the entropy feature (29). Sutton et al. extracted the first order and GLCM texture features from 178 IDC cancers to distinguish three subtypes, including luminal-like, HER2+, and TN (30). Their study analyzed only one slice showing the largest lesion diameter on the DCE images, whereas the necrotic tissue was excluded in Waugh's study. Agner et al. used the morphologic and texture features extracted from the whole tumor on the early postcontrast images in conjunction with an SVM classifier, to yield an area under the curve (AUC) of 0.74 for the TN vs. non-TN cancers (14). In our study, whole-tumor texture features extracted from DCE and DWI-related original images and those changing over six time points or three *b*-values yielded the best accuracy of 72.4% for the 4-IHC classification task, and the best accuracy of 91.0% for the TN vs. non-TN cancers. One possible interpretation for our model performance is that texture features capture the information of whole-tumor about perfusion, diffusion, and heterogeneity.

**Figure 7:**
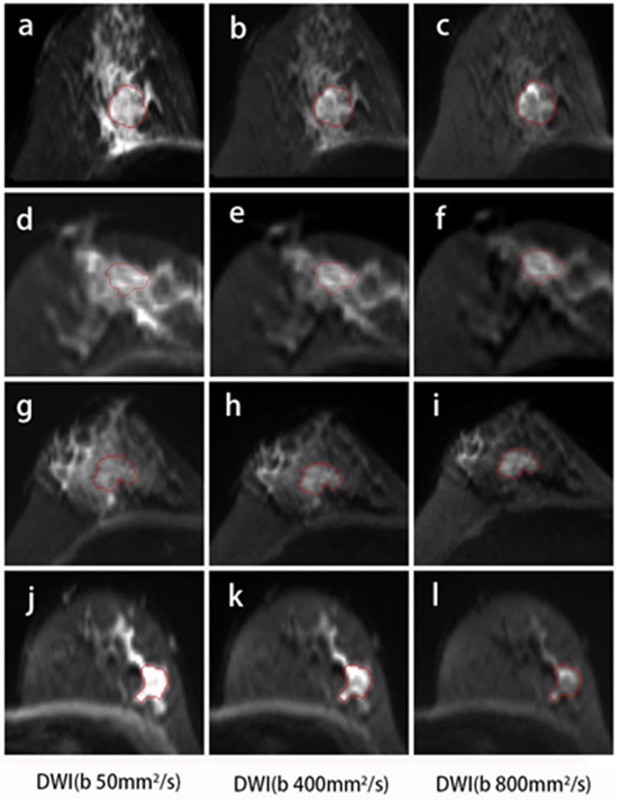
DWI images for the four subtypes of breast cancer. A 46-year-old female with Luminal A breast cancer **(A–C)**; a 60-year-old female with Luminal B breast cancer **(D–F)**; a 47-year-old female with HER2-positive breast cancer **(G–I)**; and a 57-year-old female with TN breast cancer **(J–L)**. The small dependence emphasis of Kendall-tau-b for DWI_sequential_ for Luminal A, Luminal B, HER2-positive, and TN breast cancers were −0.915, 0.358, 0.915, and 0.299, respectively.

Compared with DCE, DWI was relatively less frequently performed in the radiomics workflow due to a lower resolution and more distortion. DWI reflecting the microenvironment of tumor structures was introduced to be a complementary tool that provided additional tissue information (31). Most studies used the histogram or texture analysis on ADC maps with a mono-exponential fit (15, 32, 33). In addition, there is one study for which the authors performed histogram analysis on maps from mono-exponential DWI and biexponential intravoxel incoherent motion (IVIM) to predict the HER2 status in ER-positive breast cancer (33). These parametric maps were calculated based on different diffusion models. The applicability of several diffusion models in clinical practice remains controversial. Instead of maps, the original DWI images were used to extracted features in our study, and classification using only minor dependence emphasis on Kendall-tau-b for DWI_sequential_ yielded an accuracy of 53.7% for the 4-IHC classification. Furthermore, an accuracy of 83.6% for the TN vs. non-TN cancers was obtained using the maximum of variance for DWI_sequential_ alone. Such a finding may indicate that TN breast cancer was more heterogeneous compared with other subtypes. A possible explanation for the findings was that TN demonstrated more necrosis (34). In our study, the DWI images with three *b*-values were obtained using a 16-channel breast coil and relatively accurate *b*-values (35, 36). The features extracting for DWI may therefore offer a new strategy for reconsidering the role of DWI in radiomics. Examples of the four IHC subtypes are shown in Figure 7.

Multiple studies performed texture analysis on images from different systems and institutions, including varied protocols (14, 29, 37). The breast MR imaging protocol has a variety of acquisition parameters, such as spatial resolution and temporal resolution, dynamic time points, repetition time and echo time, *b*-values, numbers of average and acceleration factors. All these variables affect texture features, and consequently the robustness of the radiomics classification model. For the widespread use of texture analysis in clinical applications, the approach to overcome the limitation that texture features are sensitive to the imaging parameters and modalities should be considered (38). In this investigation, we extracted radiomic features from patients on both the DCE and DWI images with two new sequential images, including DCE images changing over several time points, and DWI images changing over different *b*-values, which have not been used or described before in the domain of breast radiomics. Further studies on whether these normalized features minimized the variations introduced by different reconstruction algorithms and scanning parameters between different MR vendors and institutions will be validated.

Furthermore, a novel two-stage method of feature selection was performed by combining traditional statistics with machine learning. The coarse feature selection aimed to choose a relatively small subset of the features by removing the present features of redundant, noisy, and irrelevant dimensions (39, 40). Because of the differing performance of lasso regression, ridge regression, and elastic net, we combined the three methods to select intersecting features for robustness and stability during the fine feature selection step (41). The number of fine features selected in models depends on the difficulty of the classification problem (42). In addition, the classification accuracy obtained by combining the three methods was higher compared with that obtained by using any single one of these three methods. Similarly, Li et al. combined the Mann-Whitney *U*-test and SVM to select informative genes (43).

This study had several limitations. First, it was a retrospective, single-institution study. Second, the limited sample size and the imbalance distribution of breast subtypes may not have trained the classification models sufficiently in such a retrospective design. Third, the same data were used for training and testing. The model may perform differently if multicenter datasets were used. Further prospective study using a larger and more balanced population from multiple centers and a subset of the data as a validation set is required. Fourth, the number of *b*-values for DWI to assess the texture features based on DWI image change over multiple *b*-values, was relatively low. Further validation on original DWI with more *b*-values in the IVIM (44) or diffusion kurtosis imaging (45) are needed. Finally, portions of the current work were performed offline. To implement this method into routine clinical practice, the automatic registration and segmentation tools, as well as the radiomics tools, will need to be integrated in the same platform.

In conclusion, we have shown that machine learning-based analysis of multiparametric MR radiomics assessing biological characteristics and heterogeneity of the whole tumor can effectively differentiate different subtypes of breast cancer and identify TN cancers. This approach could serve as a more convenient and non-invasive biomarker for the prediction of breast cancer subtypes.

## Data Availability

The datasets for this manuscript are not publicly available because the data of texture features and subtypes for all the patients in our study was uploded in the Supplementary Table 1.

The raw images for patients can not allowed to upload for the public access. Requests to access the datasets should be directed to pengweijun2017@163.com.

## Ethics Statement

This retrospective study was approved by the review board of Fudan University Shanghai 96 Cancer Center, which waived informed consent.

## Author Contributions

TX, ZW, WP, and HW conceived and designed the experiments. TX, ZW, QZ, YG, and HW analyzed the imaging data. QB and XZ reviewed the molecular data. TX wrote the manuscript. All authors read and approved the final manuscript.

### Conflict of Interest Statement

The authors declare that the research was conducted in the absence of any commercial or financial relationships that could be construed as a potential conflict of interest.
