# Supplementary material for: Machine Learning-Based Analysis of MR Multiparametric Radiomics for the Subtype Classification of Breast Cancer
Source: Front Oncol. 2019 Jun 14;9:505. doi: 10.3389/fonc.2019.00505 (PMC6587031; doi:10.3389/fonc.2019.00505)
Supplement: Supplementary file 2 [file Data_Sheet_1.docx]

Supplementary Material

**Supplementary material 1. Formulas for the ten new sequential features**

| p denotes patient; t denotes the phase for the DCE or DWI image with one b-value. |
| --- |
| 1. $\mathbf{Mean}=\frac{1}{N_{t}}\sum_{t=1}^{N_{t}} x_{pt}$ |
| 1. **V**$\mathbf{ariance}=\frac{1}{N_{t}}\sum_{t=1}^{N_{t}} {{(x}_{pt}-\bar{x}_{p\cdot})}^{2}$ |
| 1. $\mathbf{Skewness}=\frac{\frac{1}{N_{t}}\sum_{t=1}^{N_{t}} {(x_{pt}-\bar{x}_{p\cdot})}^{3}}{\left( \sqrt{\frac{1}{N_{t}}\sum_{t=1}^{N_{t}} {(x_{pt}-\bar{x}_{p\cdot})}^{2}} \right)^{3}}$ |
| 1. $\mathbf{Kurtosis}=\frac{\frac{1}{N_{t}}\sum_{t=1}^{N_{t}} {(x_{pt}-\bar{x}_{p\cdot})}^{4}}{\left( \sqrt{\frac{1}{N_{t}}\sum_{t=1}^{N_{t}} {(x_{pt}-\bar{x}_{p\cdot})}^{2}} \right)^{2}}$ |
| 1. $\mathbf{Energy}=\sum_{t=1}^{N_{t}} {x_{pt}}^{2}$ |
| 1. $\mathbf{Entropy}=-\sum_{t=1}^{N_{g}} p_{t}{log}_{2}\left( p_{t}+\epsilon\right)$  - $P_{t}$ is the first order histogram with $N_{g}$ discrete intensity levels, where $N_{g}$ is the number of non-zero bins - $p_{t}$ is the normalized first order histogram and is equal to $\frac{P_{t}}{N_{t}}$. - Here, ϵ is an arbitrarily small positive number (≈2.2×${10}^{-16}$). |
| 1. **Kendall's tau-b**   Kendall's tau-b is a nonparametric measure of association based on the number of concordances and discordances in the paired observations. In this work, Kendall's tau-b was used to measure the coordinated consistency of changing features between one patient and the remaining patients.  The consistent coefficient between two patients (p and p’) at two time points (t and t’) was calculated as follows (Equation (1)):  $I=\frac{x_{p't'}-x_{pt'}}{x_{p't}-x_{pt}} (1)$ (p=1, 2…,$p_{t}$, p’=p+1, p+2…,$p_{t}$ , t=1, 2…,$N_{t}$ , t’=t+1, t+2…,$N_{t}$,)  where x is the value of one of the texture features, $N_{p}$ is the number of patients, and $N_{t}$ is the number of time points. Therefore, $N_{t}$*($N_{t}$-1)/2 consistent coefficients, in total, were obtained for each pair of patients.  The consistent coefficients are subsequently subclassified into four groups: $I>0, I<0, I=0, and I\to\infty$. The corresponding number of items is: $N_{I>0},N_{I<0},N_{I=0}$ , and $N_{I\to\infty}$ .  Kendall's tau-b was calculated as follows:  $\tau=\frac{N_{I>0}-N_{I<0}}{\sqrt{(N_{I>0}+N_{I<0}+N_{I=0})\times(N_{I>0}+N_{I<0}+N_{I\to\infty})}} (2)$ |
| 1. **Conservation**   The conservation of patients was calculated as follows:  $r_{pp'}=\left\vert\frac{N_{t}\sum_{t=1}^{N_{t}} x_{pt}x_{p't}-\sum_{t=1}^{N_{t}} x_{pt}\sum_{t=1}^{N_{t}} x_{p't}}{\sqrt{N_{t}\sum_{t=1}^{N_{t}} x_{pt}^{2}-{(\sum_{t=1}^{N_{t}} x_{pt})}^{2}}\sqrt{N_{t}\sum_{t=1}^{N_{t}} x_{p't}^{2}-\left( \sum_{p=1}^{N_{t}} x_{p't} \right)^{2}}} \right\vert(3)$  Here, the absolute value of the Pearson correlation coefficient was used because both positive and negative values indicate that the two patients are relatively conserved. |
| 1. **Stability**   Stability was defined by Equation (4):  $S_{pp'}=1-\max\left\{ {PCV}_{p},{PCV}_{p} \right\} (4)$  where ${PCV}_{p}$(or ${PCV}_{p'}$) indicates the percentage of patients whose coefficient of variation (CV) did not exceed the CV of patient $p$ (or $p'$).  ${PCV}_{p}=\frac{number of patients with CV<{CV}_{p}}{total number of patinets(N_{p})} (5)$ |
| 1. **Dispersion**   Let $\mathrm{Max}_{p}=max(x_{p1},x_{p2},\ldots,x_{pT})$,$\mathrm{Min}_{p}=min(x_{p1},x_{p2},\ldots,x_{pT})$,$\mathrm{Max}_{p'}=\max\left( x_{p'1},x_{p'2},\ldots,x_{p'T} \right)$, $\mathrm{Min}_{p'}=\min\left( x_{p'1},x_{p'2},\ldots,x_{p'T} \right)$.  We assumed that $\mathrm{Max}_{p}$ was larger than $\mathrm{Max}_{p'}$, and the dispersion was defined as follows:  $D_{pp'}=\left\{ \begin{aligned} 0 if {Max}_{p}\geq{Max}_{p'}\geq{Min}_{p'}\geq{Min}_{p} \\ 1-\frac{{Max}_{p}'-{Min}_{p}}{{Max}_{p}-{Min}_{p^{'}}} if {Max}_{p}\geq{Max}_{p'}\geq{Min}_{p}\geq{Min}_{p'} \\ 1 if {Max}_{p}\geq{Min}_{p}\geq{Max}_{p'}\geq{Min}_{p'} \end{aligned} \right.$ (6) |

**Supplementary material 2. Twenty selected features for 4-IHC classification task**

| Number | Image Set | Feature Type | Feature | Definition of the Feature |
| --- | --- | --- | --- | --- |
| X1 | DWI_b800_ | GLRLM | Long Run Low Gray Level Emphasis | the joint distribution of long run lengths with lower gray-level values |
| X2 | DWI_sequential__Variance | First Order | Median | the median gray level intensity within the ROI |
| X3 | DWI_sequential__Variance | GLCM | Maximum Probability | the occurrences of the most predominant pair of neighboring intensity values |
| X4 | DWI_sequential__Variance | GLSZM | Size-Zone Non-Uniformity Normalized | the variability of size zone volumes throughout the image |
| X5 | DWI_sequential__Variance | GLSZM | Small Area Low Gray Level Emphasis | the proportion in the image of the joint distribution of smaller size zones with lower gray-level values |
| X6 | DWI_sequential__Skewness | GLDM | Dependence Entropy | the entropy in dependence size in the image |
| X7 | DWI_sequential__Kendall-tau-b | GLDM | Small Dependence Emphasis | the distribution of small dependencies |
| X8 | DWI_sequential__Conservation | GLSZM | Small Area Emphasis | a measure of the distribution of small size zones |
| X9 | DWI_sequential__Dispersion | GLDM | Large Dependence Low Gray Level Emphasis | the joint distribution of large dependence with lower gray-level values |
| X10 | DWI_sequential__Dispersion | GLSZM | Small Area Low Gray Level Emphasis | the proportion in the image of the joint distribution of smaller size zones with lower gray-level values |
| X11 | DCE_1^st^ postcontrast phase | GLSZM | Zone Entropy | the uncertainty/randomness in the distribution of zone sizes and gray levels |
| X12 | DCE_sequential__Conservation | NGTDM | Coarseness | a measure of average difference between the center voxel and its neighbourhood and is an indication of the spatial rate of change |
| X13 | DCE_sequential__Dispersion | GLCM | Informational Measure of Correlation (IMC) 1 | the correlation between the probability distributions (quantifying the complexity of the texture) |
| X14 | DCE_sequential__Variance | First Order | 10^th^ Percentile | the 10th percentile of intensity values |
| X15 | DCE_sequential__Skewness | GLSZM | Size-Zone Non-Uniformity | the variability of size zone volumes in the image |
| X16 | DCE_sequential__Skewness | GLSZM | Size-Zone Non-Uniformity Normalized | the variability of size zone volumes throughout the image |
| X17 | DCE_sequential__Energy | GLCM | Informational Measure of Correlation (IMC) 1 | the correlation between the probability distributions (quantifying the complexity of the texture) |
| X18 | DCE_sequential__Entropy | GLCM | Inverse Difference Moment (IDM) | a measure of the local homogeneity of an image |
| X19 | DCE_sequential__Entropy | GLSZM | Gray Level Non-Uniformity | the variability of gray-level intensity values in the image |
| X20 | DCE_sequential__Entropy | GLSZM | Small Area Emphasis | a measure of the distribution of small size zones |

DWI = diffusion-weighted imaging, DCE = dynamic contrast-enhanced, GLCM = gray level co-occurrence matrix, GLRLM = gray level run length matrix, GLSZM = gray level size zone matrix, GLDM = gray level dependence matrix, NGTDM = neighboring gray tone difference matrix, IHC= Immunohistochemical.

**Supplementary material 3. Eight selected features for TN vs. non-TN cancers**

| Number | Image Set | Feature Type | Feature | Definition of the Feature |
| --- | --- | --- | --- | --- |
| Y1 | DWI_b800_ | First Order | Skewness | the asymmetry of the distribution of values about the Mean value. |
| Y2 | DWI_sequential__Variance | First Order | Maximum | the maximum gray level intensity |
| Y3 | DWI_sequential__Energy | NGTDM | Strength | a measure of the primitives in an image |
| Y4 | DWI_sequential__Conservation | First Order | Median | the medium gray level intensity |
| Y5 | DWI_sequential__Conservation | GLSZM | Small Area Emphasis | a measure of the distribution of small size zones |
| Y6 | DWI_sequential__Conservation | NGTDM | Strength | a measure of the primitives in an image |
| Y7 | DCE_sequential__Entropy | GLCM | Difference Average | the relationship between occurrences of pairs with similar intensity values and occurrences of pairs with differing intensity values |
| Y8 | DCE_sequential__Entropy | GLSZM | Gray Level Non-Uniformity | the variability of gray-level intensity values in the image |

DWI = diffusion-weighted imaging, DCE = dynamic contrast-enhanced, GLCM = gray level co-occurrence matrix, GLSZM = gray level size zone matrix, GLSZM = gray level size zone matrix, NGTDM = neighboring gray tone difference matrix, TN= triple negative.

**Supplementary material 4. The Rad-score Formulas for 4-IHC Classification**

Rad-score_1_ = 2.26870 - 0.23865 * X1 + 0.21846 * X2 + 0.27868 *X3 – 0.15692 * X4 + 0.16510 * X5 – 0.17217 * X6 – 0.15263 * X7 + 0.32341 * X8 - 0.11407 * X9 + 0.33683 * X10 + 0.19851* X11 - 0.14929 * X12 - 0.39553 * X13 - 0.17860 * X14 + 0.17461 * X15 - 0.27390 * X16 - 0.17488 * X17 + 0.24216 * X18 - 0.25105 * X19 + 0.03560 * X20

**Supplementary material 5. The Rad-score Formulas for TN vs. non-TN cancers**

Rad-score_2_ = 0.16420 - 0.08676 * Y1 + 0.09858 * Y2 + 0.10960 * Y3 - 0.07453 * Y4 - 0.09582 * Y5 - 0.07397 * Y6 - 0.13334 * Y7 + 0.08432 * Y8
